# A Transient Auditory Signal Shifts the Perceived Offset Position of a Moving Visual Object

**DOI:** 10.3389/fpsyg.2013.00070

**Published:** 2013-02-21

**Authors:** Sung-en Chien, Fuminori Ono, Katsumi Watanabe

**Affiliations:** ^1^Research Center of Advanced Science and Technology (Cognitive Science), The University of TokyoMeguro-ku, Tokyo, Japan; ^2^Yamaguchi UniversityYamaguchi-shi, Yamaguchi, Japan

**Keywords:** motion offset, audiovisual interaction, representational momentum, visual motion representation, auditory transients

## Abstract

Information received from different sensory modalities profoundly influences human perception. For example, changes in the auditory flutter rate induce changes in the apparent flicker rate of a flashing light (Shipley, 1964). In the present study, we investigated whether auditory information would affect the perceived offset position of a moving object. In Experiment 1, a visual object moved toward the center of the computer screen and disappeared abruptly. A transient auditory signal was presented at different times relative to the moment when the object disappeared. The results showed that if the auditory signal was presented before the abrupt offset of the moving object, the perceived final position was shifted backward, implying that the perceived visual offset position was affected by the transient auditory information. In Experiment 2, we presented the transient auditory signal to either the left or the right ear. The results showed that the perceived visual offset shifted backward more strongly when the auditory signal was presented to the same side from which the moving object originated. In Experiment 3, we found that the perceived timing of the visual offset was not affected by the spatial relation between the auditory signal and the visual offset. The present results are interpreted as indicating that an auditory signal may influence the offset position of a moving object through both spatial and temporal processes.

## Introduction

Tracking the trajectory and localizing the position of a moving visual object are essential abilities for carrying out many tasks in everyday life. Studies have demonstrated that the perceived or remembered position of a moving object is consistently biased in the forward direction of motion. This forward bias is referred as representational momentum (RM) which can be observed in both implied and continuous motion. Studies of RM have also demonstrated that the final perceived position of a moving object is mislocalized in the forward direction of motion (Freyd and Finke, 1984; Hubbard and Bharucha, 1988). RM could result from the mental representation of the object’s motion persisting for a brief period after abrupt offset (Teramoto et al., 2010).

The perceptual system receives information through different, interacting sensory modalities. The inputs from different sensory modalities interact in various ways. In this study, we were interested in whether the perceived position of a visual motion offset would be influenced by a transient auditory signal.

Several previous studies have investigated how visual motion perception is modulated by a transient auditory signal. In the flash-lag effect, the perceived position of a moving object appears to be relatively ahead of a physically aligned flash (e.g., Nijhawan, 1994; Watanabe and Yokoi, 2006, 2007, 2008; Maus and Nijhawan, 2009). This phenomenon seems to be a result of the visual representation of moving objects being spatially shifted forward to counteract delays in the neural system on the perceived position. Vroomen and de Gelder (2004) showed that the magnitude of the flash-lag effect is reduced when a transient auditory signal is presented before or simultaneously with the flash. In addition, Heron et al. (2004) demonstrated that the location of a horizontally moving object that changes its direction against a vertical virtual surface is perceptually displaced forward with respect to the direction of previous motion when a sound is presented after the actual bounce event, and the perceived bounce position is shifted in the direction opposite to previous motion when a sound is presented before the actual bounce. Fendrich and Corballis (2001) asked participants to report the position of a rotating flash when an audible click was heard. The flash was seen earlier when it was preceded by an audible click and later when followed by the click.

These studies indicate the possibility that, when judging the offset position of a moving visual object, our perceptual system may not rely exclusively on visual information, but may also utilize information from other modalities. However, this explanation is not completely consistent with the modality precision hypothesis, which suggests that the modality with the highest precision with regard to the required task tends to be dominant in multimodal interactions (Shipley, 1964; Welch and Warren, 1980, 1986; Welch et al., 1986; Spence and Squire, 2003). The modality precision hypothesis would suggest that when judging the offset position of a moving visual object, the perceived visual offset would be processed exclusively by the visual system rather than also utilize information from other modalities (e.g., audition). Therefore, we hypothesized that in a situation allowing a transient auditory stimulus to be associated with a visual motion offset, the auditory stimulus will influence the perceived final position of the moving object.

Recently, Teramoto et al. (2010) found that the magnitude of RM is influenced by a continuous sound accompanying a moving visual object. They showed that RM is enhanced when the sound terminates after the offset of the visual object, but reduced when the sound terminates before visual offset. However, their results also indicated that transient auditory signals presented at the onset and around the offset of the visual motion had no effect on the perceived offset position of the visual object. On the basis of these observations, they suggest that the sustained sound during visual motion is necessary for the audiovisual integration to have an effect. However, based on studies indicating that visual motion perception can be modulated by a transient auditory signal (Fendrich and Corballis, 2001; Heron et al., 2004; Vroomen and de Gelder, 2004), it is still possible that the visual offset position could be influenced when a transient sound is presented temporally proximal to the offset of the visual stimulus without an auditory signal having been presented at the motion onset. Additionally, in the study by Teramoto et al. (2010), the authors measured RM with a probe-judgment task. However, a mouse-pointing task is typically used with continuous motion target (Hubbard, 2005). In light of this information, we decided to measure the perceived visual offset position by using a mouse-pointing task in the present study.

Multisensory interactions are also affected by the characteristics of the stimuli in different modalities. For example, a single visual flash can be perceived as multiple flashes if accompanied by multiple auditory stimuli (sound-induced illusory flash). Discontinuous stimuli in one modality seem to alter the perception of continuous stimuli in another modality. This indicates that multisensory interaction is at least partly affected by stimulus characteristics: continuous versus discontinuous (Shams et al., 2002). Additionally, Courtney et al. (2007) reported that one flash presented near a visual fixation induces an illusory flash in the periphery. Courtney et al. suggest that the effect of stimulus discontinuity/continuity may also be valid for unisensory stimuli.

The multisensory effect of a transient stimulus is not confined to perceptual alternation between competing incompatible interpretations when the perceptual system is confronted with ambiguous stimuli. The multisensory effect can also be observed when there are no competing incompatible interpretations. Attentional repulsion is described as the perceived displacement of a vernier stimulus in a direction that is opposite to a brief peripheral visual cue. Arnott and Goodale (2006) demonstrated that the repulsion effect could be induced by presenting lateralized sounds as peripheral cues, showing that auditory spatial information can displace the perceived positions of static visual stimuli. This finding indicates the possibility that the location of sound may affect the retinotopic coding. Recently, Teramoto et al. (2012) presented results of a study of visual apparent motion in conjunction with a sound delivered alternately from two loudspeakers aligned horizontally or vertically. Participants reported that the direction of visual apparent motion was consistent with the direction of sound alternation or the auditory stimulus influenced the path of apparent motion. The researchers suggest that auditory spatial information could also modulate the perception of a visual moving object, especially in the peripheral visual field.

Audiovisual interaction is enhanced when visual signals and auditory signals are presented in close proximity spatially. For example, observers are more likely to report that visual stimuli and auditory stimuli are presented simultaneously when they originate from the same spatial position than when they originate from different positions (Zampini et al., 2005). When observers are asked to determine the direction of auditory apparent motion while trying to ignore unrelated visual motion, they perform worse when the auditory motion is in the opposite direction to the visual apparent motion. This audiovisual dynamic capture effect is larger when the auditory and visual stimuli are presented from close spatial locations (Soto-Faraco et al., 2002; Meyer et al., 2005; Spence, 2007).

On the basis of these findings, we hypothesized that it is possible for auditory information to affect perceived visual motion offset in the peripheral visual field, and that this effect will be enhanced when visual stimuli and auditory stimuli are presented to the same hemifield. Because studies have indicated that an auditory transient can alter apparent motion perception (e.g., Heron et al., 2004), we examined whether a transient auditory signal would affect the perceived offset position of a visual moving object, and if so, spatial contingency between auditory signal and visual object would enhance the auditory modulation. To achieve this goal, we presented a transient sound around the time of visual motion offset and asked participants to report the perceived offset position of the visual stimulus (Experiment 1). In addition, we tested whether the auditory spatial information would influence the effect of the auditory stimulus on the perceived visual offset position (Experiment 2). After affirmative results were obtained in both experiments, we examined whether the auditory effects were caused by distortion in the perceived timing of the offset of the visual moving object (Experiment 3).

## Experiment 1

In Experiment 1, we examined the possibility that the timing of a transient auditory signal would affect the perceived offset position of a visual moving object. Such an effect would demonstrate that a continuous auditory stimulus during visual motion is not necessary to alter the perceived visual offset position. We conducted Experiment 1A and 1B. The visual target appeared in left visual field and moved rightward (Experiment 1A) or in right visual field and made rightward motion (Experiment 1B), and then the visual target disappeared around the center the display. A transient auditory signal was presented around the visual motion offset of the visual target. We treated the two motion direction conditions as a between-subjects variable to reduce task loads for each participant.

### Method

#### Participants

There were 16 paid volunteers in Experiment 1A (10 males, 6 females) and 1B (11 males, 5 females). Their ages ranged from 20 to 34 years (mean = 25.1) in Experiment 1A and from 19 to 28 years (mean = 21.7) in Experiment 1B. All were right-handed by self-report. All participants had normal or corrected-to-normal vision and audition and were naïve as to the purpose of this study.

#### Apparatus and stimuli

**Figure 1:**
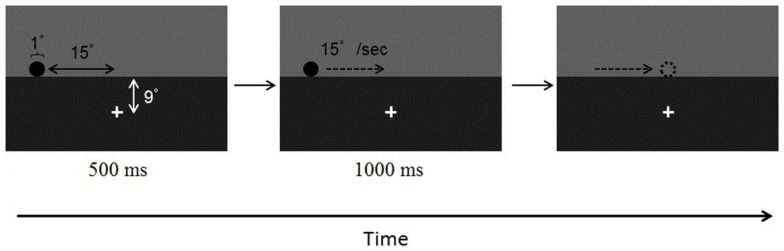
**Example of the visual display in Experiment 1**.

Participants observed the visual stimuli on a 23′′ CRT monitor at a viewing distance of 60 cm. The monitor’s refresh rate was 100 Hz. The visual and auditory stimuli were presented using the MATLAB operating environment and the Psychtoolbox extensions (Brainard, 1997; Pelli, 1997). The background was divided horizontally into two parts (Figure 1). The upper part was gray (40° × 10.5°, 7.85 cd/m^2^) and the lower part was black (40° × 19.5°, 0.03 cd/m^2^). A white fixation cross (1° × 1°, 61.27 cd/m^2^) was presented at the center of the lower background.

The visual stimulus was a black disk (1° in diameter) that appeared at the bottom of the gray background, 15° to the left (Experiment 1A) or right (Experiment 1B) of the midpoint. The disk moved from left to right (Experiment 1A) or from right to left (Experiment 1B) at a constant speed of 15°/s. The disk disappeared when its center was at the midpoint or randomly jittered from the midpoint by ±0.3°. The auditory stimulus was a transient auditory signal with a 1000-Hz pure tone without onset or offset intensity ramps, presented via headphones to both ears for 10 ms. Note that previous research has shown that a 10-ms-sound could produce effect on audio-visual interaction (e.g., Fujisaki et al., 2004; Ono and Kitazawa, 2011). The approximate range of sound pressure level was 60–65 dB. The sound was presented 120, 80, or 40 ms before the visual motion offset, simultaneously with the visual offset (0 ms), or 40, 80, or 120 ms after the visual offset. As a control condition, we included trials in which the sound was absent.

#### Procedure

Participants started each trial by pressing the space key. The black disk appeared and stayed stationary at the initial position for 500 ms. Participants were asked to observe the disk while keeping their eyes on the fixation cross. After the initial stationary period, the black disk moved at a constant speed of 15°/s for 1000 ms and then disappeared around the midpoint of the display. A mouse cursor appeared 1° above the fixation cross 200 ms after the disappearance of the visual target. The participants were instructed to move the mouse cursor and click the mouse button at the target’s visual offset position.

Participants performed 10 practice trials to familiarize themselves with the position judgment task. Then, they performed 10 trials in each combination of conditions for a total of 240 trials (8 sound conditions × 3 visual offset positions × 10 trials). Trials of all conditions were randomly ordered.

#### Statistical analysis

The data were submitted to a two-way mixed-design analysis of variance (ANOVA) followed by *post hoc* comparisons with the *Bonferroni* correction with the alpha level set at 0.05.

### Results and discussion

**Figure 2:**
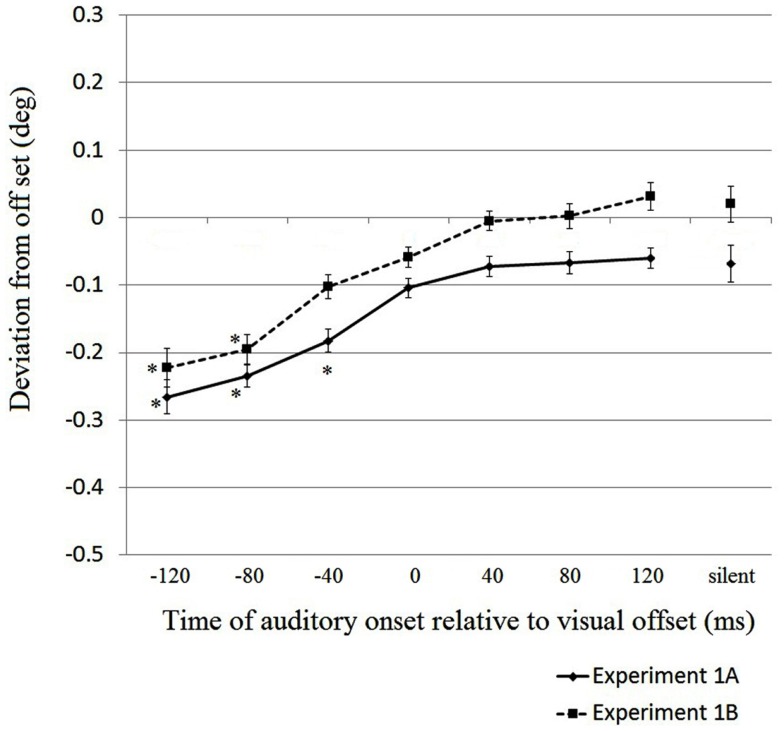
**Results of Experiments 1A and 1B**. The horizontal axis represents the experimental conditions for different presentation timings of the auditory signal. The vertical axis represents the perceived deviation from the physical visual offset position in degrees of visual angle. A negative value in the deviation from offset (*Y*-axis) means that the perceived visual offset position was behind the actual visual offset position. Error bars represent within-participants SEMs (Loftus and Masson, 1994; Cousineau, 2005) for each presentation. Data points with an * mark indicate that the perceived positions differ from 0.

We calculated the average deviation of the perceived visual offset position from the physical visual offset point for each sound condition. Figure 2 shows the combined results of Experiments 1A and 1B. The horizontal axis represents the different sound conditions. The vertical axis represents the perceived deviation from the actual physical visual offset position. A negative value in the deviation from visual offset (*Y*-axis) means that the perceived visual offset position was behind the actual visual offset position.

We performed a two-way mixed-design ANOVA, in which the visual field of start position was the between-subjects factor and the timing of the auditory signal was treated as the within-subject factor. The main effect of the visual field of start position was not significant [*F*(1,30) = 0.499, *p* = 0.485]. The main effect of the timing of the auditory signal was significant [*F*(7,210) = 36.261, *p* < 0.001]. There was no significant interaction between the visual field of start position and the timing of the auditory signal [*F*(7,210) = 0.48, *p* = 0.849]. Overall, these results suggest that the earlier the auditory signal was presented, the farther away the visual offset was shifted backward (i.e., the perceived visual offset position shifted backwards).

Then, we compared the cell means of the perceived visual offset position against zero to test whether there was a significant displacement from the actual position in each condition (Table 1). The adjusted alpha level was 0.006 (0.05/8) when comparing the cell means against zero. In Experiment 1A, only the −120, −80, and −40 ms conditions significantly differed from zero [*t*(15) = 5.69, *t*(15) = 5.88, and *t*(15) = 5.89, respectively; all *p* < 0.006]. In Experiment 1B, the −120 and −80 ms conditions differed significantly from zero [*t*(15) = 6.57 and *t*(15) = 5.27, respectively; *p* < 0.006]. Thus, we confirmed that when the auditory signal was presented before the physical offset of the visual stimulus, the visual offset position tended to be perceived as behind the actual physical visual offset position. Conversely, no significant displacement was found in the 0, 40, 80, and 120 ms conditions, implying that the auditory signal did not produce an effect when presented after or at the moment of the visual motion offset.

**Table 1:** **Perceived offset position in Experiment 1 in visual degree**.

	−120	−80	−40	0	40	80	120	Silent
Experiment 1A	−0.266*	−0.234*	−0.182*	−0.104	−0.072	−0.067	−0.060	−0.068
Experiment 1B	−0.222*	−0.196*	−0.102	−0.059	−0.005	0.002	0.031	0.020

Values with * mark indicated that perceived displacements significantly differ from zero.

We also compared the cell means of each condition in which an auditory signal was presented to the cell mean of the silent condition. The adjusted alpha level is 0.007 (0.05/7). In Experiment 1A, the perceived visual offset positions in the −120, −80, and −40 ms conditions differed from that in the silent condition [*t*(15) = 4.46, *t*(15) = 4.23, and *t*(15) = 3.34, respectively; all *p* < 0.007]. In Experiment 1B, the perceived visual offset positions in the −120, −80, and −40 ms conditions differed from that in the silent condition [*t*(15) = 5.24, *t*(15) = 5.01, and *t*(15) = 3.30, respectively; all *p* < 0.007]. We observed that the silent condition did not differ from the conditions in which the auditory signal was presented after physical visual offset in either Experiment 1A [*t*(15) < 1.11, *p* > 0.05] or 1B [*t*(15) < 1.05, *p* > 0.05].

The lack of RM in the present experiments is notable, but similar findings have been reported in several previous studies in which observers were given instructions to maintain fixation. Previous research has also indicated that fixation decreases RM for targets with smooth and continuous motion (Kerzel, 2000). It is possible that we did not observe RM in Experiment 1 because we used visual stimuli with smooth and continuous motion. However, RM has also been observed for targets with implied motion and for frozen-action photographs that do not elicit eye movements (Kerzel, 2003; Hubbard, 2005, 2006). Although we emphasized to participants the importance of maintaining focus on the fixation cross, we did not record eye movements. In order to examine whether eye movements might have played a major role in the present experiment, we performed an experiment for a supplementary examination using the same stimuli as in Experiment 1A, in which participants (*N* = 5) were free to move their eyes during the experiment. The results showed the same pattern as Experiment 1A [*F*(7,28) = 8.028, *p* < 0.001]. We observed a tendency toward greater backward displacement when the sound was presented earlier. Therefore, the lack of RM in the present study cannot be explained completely by the instruction to maintain fixation. The lack of RM might be due partially to the shorter delay from the target offset to the appearance of the mouse cursor. Kerzel et al. (2001) showed that RM was larger with the longer delay between the target and probe. The delay was 200 ms in our study while it was 500 ms in Teramoto et al.’s study.

In the present study, it is more likely that the perceived timing of visual motion offset was attracted toward the timing of the presentation of the transient sound when the sound was presented before the physical visual motion offset, which resulted in the decreased magnitude of RM and consequently induced backward displacement. When the transient sound was presented after the physical visual motion offset, the perceived visual offset position of the visual target did not differ from the condition in which the sound was absent. In addition, our results also imply that this effect might not be confined to a visual stimulus presented at the periphery that moves to the foveal region. However, these issues require further empirical examination.

We also analyzed the average response times for completing the mouse-pointing task in each trial. Response times were not affected by different auditory stimulus timings [visual field of start position, *F*(1,30) = 0.967; timing of the auditory signal, *F*(7,210) = 0.873; interaction, *F*(7,210) = 0.250; all *p* > 0.05].

## Experiment 2

In Experiment 2, we investigated whether the spatial contingency between auditory signals and visual events would modulate the auditory influence on the perceived offset position of the visual motion. We presented a lateralized transient auditory signal to either the left or the right ear with the same visual stimuli used in Experiment 1. The visual target appeared at left visual field and moved rightward in Experiment 2A. In Experiment 2B, the visual target appeared at right visual field and moved leftward. The visual field of start position was treated as a between-subjects variable to reduce the task load for each participant.

### Method

#### Participants

There were 15 paid volunteers in Experiment 2A (10 males, 5 females) and 2B (9 males, 6 females). Their ages ranged from 19 to 31 years (mean = 21.79) in Experiment 2A and from 19 to 25 years (mean = 21.72) in Experiment 2B. All participants were right-handed by self-report, except for one left-handed participant in Experiment 2B. All of the participants had normal or corrected-to-normal visual acuity and were naïve as to the purpose of this study.

#### Stimuli and procedure

The apparatus and visual stimuli of Experiment 2 were the same as those of Experiment 1 except for the following points. In Experiment 2, the auditory signals were presented to the left ear in the half of trials and to the right ear in the other half of trials. The sound was presented 40, 80, or 120 ms before or after the offset of the visual target or at the same time as the visual offset. Since we did not find any differences between any conditions in which the auditory signal was presented after physical visual offset and the silent condition in Experiment 1, we did not include a silent condition in Experiment 2. After 10 training trials, the participants performed 10 trials of each experimental condition for a total of 420 trials (2 sound positions × 3 visual offset positions × 7 timings × 10 trials). Trials of all conditions were randomly ordered, so that in each trial the auditory signal might be presented to the same or the opposite side as the visual target origination.

#### Statistical analysis

The data were submitted to a three-way mixed-design ANOVA followed by *post hoc* comparisons with *Bonferroni* corrections *p* < 0.05.

### Results and discussion

**Figure 3:**
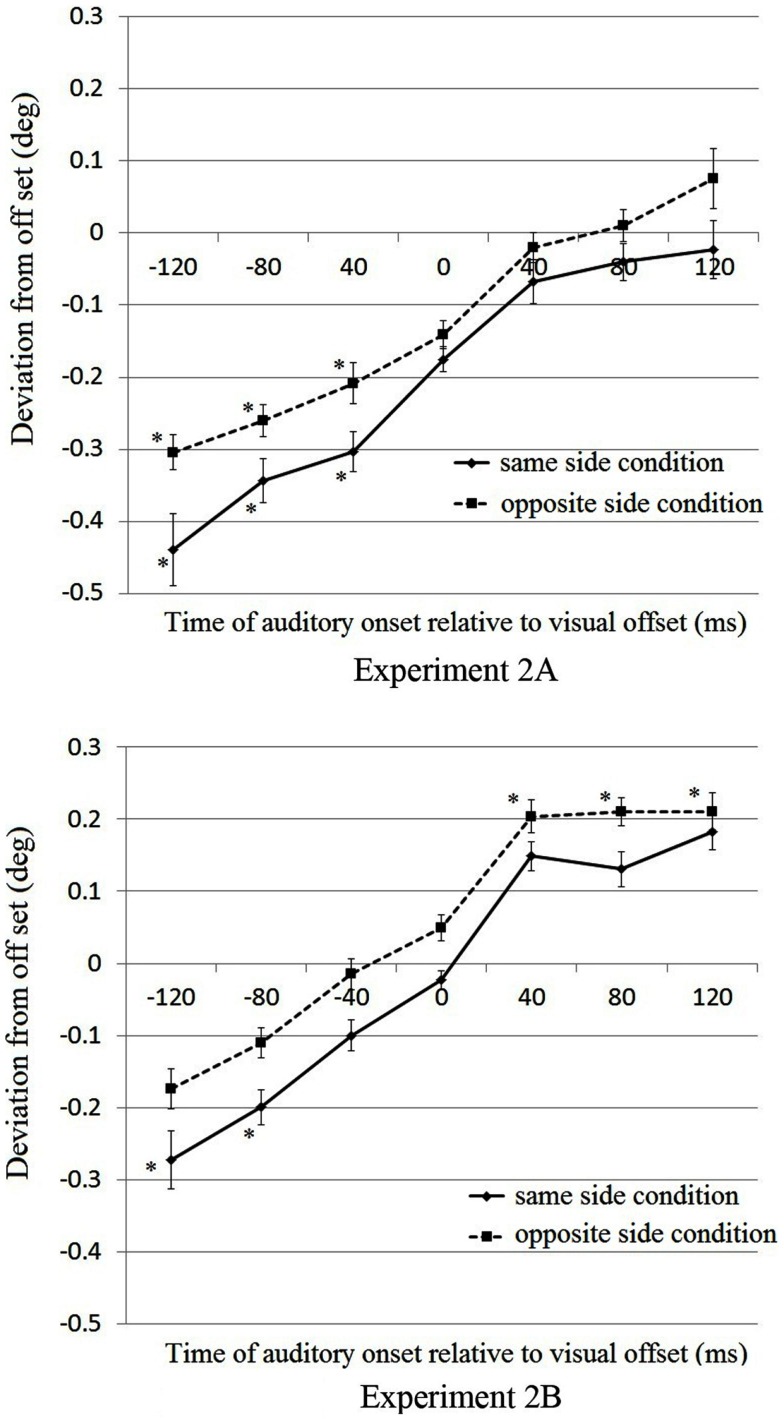
**Results of Experiments 2A (top) and 2B (bottom)**. The horizontal and vertical axes represent, respectively, the different sound presentation conditions and the perceived deviation from the physical visual offset position in degrees of visual angle. A negative value in the deviation from visual offset (*Y*-axis) means that the perceived visual offset position was behind the actual visual offset position. Error bars represent within-participants SEMs (Loftus and Masson, 1994; Cousineau, 2005) for each presentation. Data points with an * mark indicate that the perceived positions differ from 0.

The top and bottom panels of Figure 3 show the results of Experiments 2A and 2B, respectively. We conducted a three-way mixed-design ANOVA, in which the visual field of the start position was the between-subjects factor and the sound contingency and the timing of the auditory signal were treated as within-subject factors. The sound contingency indicates if the auditory signal was presented to the same or the opposite side as the originating position of the visual target. We found the significant main effects of auditory timing [*F*(6,168) = 77.48, *p* < 0.001] and sound contingency [*F*(1,28) = 43.526, *p* < 0.001]. The main effect of the start position approached the significance level [*F*(1,28) = 4.127, *p* = 0.052]. There were no significant interactions [visual field × sound contingency, *F*(1,28) = 0.049; visual field × auditory timing, *F*(6,168) = 0.52; sound contingency × auditory timing, *F*(6,168) = 1.540; visual field × sound contingency × auditory timing, *F*(6,168) = 1.010; all *p* > 0.05]. The results imply that the timing of the auditory signal affected the perceived visual offset position, replicating the findings of Experiment 1. Furthermore, when the sound is presented in the same hemifield as the visual target’s start position, the effect was enhanced (i.e., more backward displacement). In addition, the results of Experiment 2B seemed to shift positively along the *Y*-axis, suggesting the possibility that RM was generally more pronounced in Experiment 2B.

#### Experiment 2A

We compared the cell means of the perceived visual offset position in Experiment 2A against zero to test whether there was a significant displacement from the actual visual offset position in each condition (Table 2). The adjusted level of the *p*-value required for significance with *Bonferroni* correction is 0.007 (0.05/7) when comparing the cell means to zero. When the auditory signal was presented to the same side from which the visual target appeared, the perceived visual offset positions significantly differed from zero in the −120, −80, and −40 ms conditions [*t*(14) = 5.02, *t*(14) = 4.25, and *t*(14) = 4.09, respectively; all *p* < 0.007]. When the auditory signal was presented in the hemifield opposite from which the target appeared, the perceived visual offset positions significantly differed from zero in the −120, −80, and −40 ms conditions [*t*(14) = 4.72, *t*(14) = 4.04, and *t*(14) = 4.02, respectively; all *p* < 0.007].

**Table 2:** **Perceived offset position in Experiment 2 in visual degree**.

	−120	−80	−40	0	40	80	120
**EXPERIMENT 2A**							
Same-side	−0.440*	−0.343*	−0.303*	−0.175	−0.068	−0.040	−0.023
Opposite-side	−0.304*	−0.260*	−0.208*	−0.141	−0.02	−0.011	0.075
**EXPERIMENT 2B**							
Same-side	−0.272*	−0.200*	−0.1	−0.02	0.150	0.130	0.183
Opposite-side	−0.175	−0.110	−0.015	0.050	0.204*	0.211*	0.211*

Values with * mark indicate that perceived displacements significantly differ from zero.

We also compared the cell means between different sound positions in the −120, −80, and −40 ms conditions in which significant displacements were observed. The adjusted level of the *p*-value required for significance with *Bonferroni* correction was 0.017 (0.05/3). When the auditory signal was presented to the same side as the visual target origination, the backward displacement was larger [*t*(14) = 2.44 and *t*(14) = 2.51 for the −120 and −80 ms conditions, respectively; all *p* < 0.017].

#### Experiment 2B

The results of Experiment 2B were different from Experiment 2A when comparing the cell means to zero (Table 2). The adjusted level of the *p*-value required for significance with *Bonferroni* correction is 0.007 (0.05/7) when comparing the cell means to zero. In Experiment 2B, significant forward displacements (i.e., RM) were observed in the conditions with 40, 80, and 120 ms delays and with the sound presented to the side opposite the visual target origination [*t*(14) = 3.45, *t*(14) = 3.27, and *t*(14) = 3.51 for the 40, 80, and 120 ms conditions, respectively; all *p* < 0.007]. However, there was no significant difference among these three cell means (all *p* values > 0.017, the *p*-value required for significance with *Bonferroni* correction is 0.05/3 = 0.017). RM was observed when the sound was presented to the opposite side, implying that when the auditory signal was presented to the opposite side (i.e., to the side toward which the visual target moved), it attracted the offset position of the visual target, which resulted in larger forward displacements.

Conversely, significant backward displacements were observed only in the −120 and −80 ms conditions when the sound occurred on the same side as the visual target [*t*(14) = 3.25 and *t*(14) = 3.21 for the −120 and −80 ms conditions, respectively; both *p* < 0.007], but the difference between these conditions was not significant [*t*(14) = 1.91, *p* = 0.15]. Significant backward shift was observed only when the sound was presented on the same side as the visual target. It seems that RM (i.e., forward displacement) was more evident in Experiment 2B. However, similar to the results of Experiment 2A, an auditory signal presented before the physical offset of the visual object exhibited a net effect of RM and a process induced by the transient auditory signal decreasing RM or even inducing backward displacement of the perceived visual offset position in some conditions, and this effect was stronger when the auditory signal was presented to the side from which the visual target appeared. Hubbard (2005) indicated that displacement of the perceived visual offset position is influenced by multiple factors such as RM, representational gravity, and characteristics of the context. The result of the present study implied the possibility that a transient auditory signal closely associated with the visual offset also influences the perceived visual offset from the physical visual offset position.

A consistent effect of motion direction on RM has not been reported in horizontal motion (Hubbard, 2005). Several previous researchers have suggested that forward displacement of horizontally moving targets is larger in the left visual field (Halpern and Kelly, 1993; White et al., 1993). However, this conflicts with our results in which the larger RM was observed in the right visual field. Since we did not compare rightward and leftward motion within each visual field and with sounds presented from different directions, the present study could not rule out the possibility that motion direction might have influenced the enhancement of RM.

Other research has shown that the attentional mechanisms in the left hemisphere tend to distribute attentional resources within the right visual field, while the attentional mechanisms in the right hemisphere distribute attentional resources across both left and right visual fields. Therefore, there might be a slight bias of spatial attention favoring the left visual field (Mesulam, 1999). It is possible that processing speed or acuity is slightly different between left and right visual fields. However, this issue needs to be tested in future investigations.

Averaged response times for completing the mouse-pointing task in each trial were not affected by different auditory onset times in Experiment 2 [visual field of start position, *F*(1,28) = 0.057; timing of the auditory signal, *F*(6,168) = 1.646; sound contingency, *F*(1,28) = 0.403; visual field × sound contingency, *F*(1,28) = 1.080; visual field × auditory timing, *F*(6,168) = 1.741; sound contingency × auditory timing, *F*(6,168) = 1.522; visual field × sound contingency × auditory timing, *F*(6,168) = 1.344; all *p* > 0.05].

## Experiment 3

The results of Experiment 1 implied that the perceived offset position of a visual moving object shifts backward when a transient auditory signal is presented before the physical visual offset. In addition, we observed larger backward displacement when the auditory signal was presented earlier. We interpreted this finding to mean that the perceived timing of visual motion offset is attracted toward the *timing* of the presentation of transient sound, which results in a decreased magnitude of RM and induces backward displacement. Experiment 2 showed that the perceived visual offset position exhibits a larger shift induced by the spatial information relative to the visual target when the transient auditory signal is presented to the same side as the visual field from which the moving object originates. It is possible to argue that a sound presented to the same side as the visual object might be heard earlier (perhaps because attention might be biased toward the side where the visual object appeared) and consequently shift the perceived visual offset position backward more strongly (i.e., the effect is temporal). Alternatively, the spatial information of the sound relative to the visual target might shift the perceived visual offset position toward the side of the auditory signal without influencing the timing judgment (i.e., a spatial attraction of the visual offset by the auditory signal). Experiment 3 was conducted to examine if the relative timing between visual and auditory events differs when the sound is presented to the same or opposite side as the visual object. Although RM was observed only in Experiment 2B, the results of Experiments 2A and 2B were similar. For this reason, we used the same visual and auditory stimuli as in Experiment 2A, but we asked participants to perform a temporal-order judgment task.

### Method

#### Participants

Fifteen paid volunteers participated in the experiment (9 males, 6 females). Their ages ranged from 20 to 25 years (mean = 22.4) and all were right-handed. All the participants had normal or corrected-to-normal visual acuity and were naïve as to the purpose of this study.

#### Stimuli and procedures

The apparatus and stimuli were identical to those used in Experiment 2A. Participants were asked to focus on the fixation cross and observe the moving object. The transient auditory signal was presented −120, −80, −40 ms before the visual offset; synchronous with the visual offset; or 40, 80, or 120 ms after the visual offset. The participants were asked to judge whether the auditory signal was presented before or after the offset of the moving disk. After 10 training trials, 10 experimental trials in each condition were presented for a total of 420 trials (2 sound positions × 3 visual offset positions × 7 sound timings × 10 trials). Trials of all conditions were randomly ordered.

#### Statistical analysis

The data were submitted to a two-way mixed-design ANOVA.

### Results and discussion

**Figure 4:**
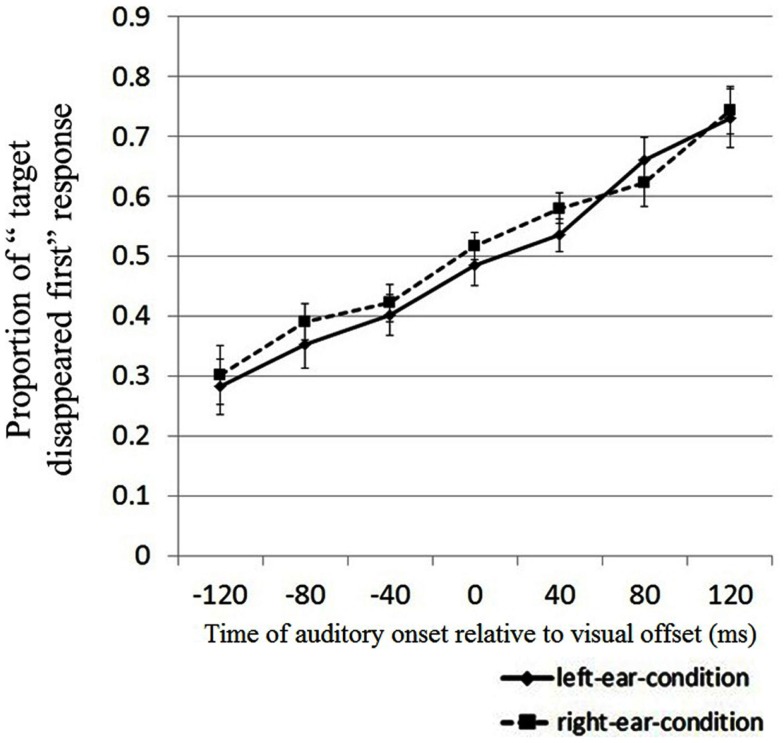
**Results of Experiment 3**. The horizontal and vertical axes represent the different sound presentation conditions and the proportion of “target disappeared first” responses, respectively. Error bars represent within-participants SEMs (Loftus and Masson, 1994; Cousineau, 2005) for each presentation.

Figure 4 shows the results of Experiment 3. A two-way repeated measures ANOVA revealed that the main effect of sound timing was significant [*F*(6,84) = 18.214, *p* < 0.001], while the main effect of sound contingency was not significant [*F*(1,14) = 0.90, *p* = 0.358]. No interaction was observed between sound timing and sound contingency [*F*(6,84) = 0.735, *p* = 0.623]. Thus, the proportion of “target disappeared first” responses increased with the delay of the auditory signal, and more importantly, the proportion of these responses did not differ between the same-side and opposite-side conditions. This suggests that the spatial information of the auditory signal did not affect the judgment of relative timing between auditory events and visual events. Therefore, enhanced displacement induced by the sound from the same visual field with the visual target in Experiment 2 resulted from the spatial information of the sound relative to the visual target. It produced a larger spatial attraction of the visual offset. The effect of a sound’s spatial information did not interact with the effect of the sound’s temporal information.

Averaged response times for completing the temporal-order judgment task in each trial were not affected by the auditory timing or sound contingency in Experiment 3 [sound contingency, *F*(1,14) = 0.852; auditory timing, *F*(6,84) = 1.229; interaction, *F*(6,84) = 1.205; all *p* > 0.05].

## General Discussion

The present study reports several novel findings. First, a transient auditory signal presented before the visual offset of a moving object shifted the perceived visual offset position backward as if it truncated the visual trajectory (Experiment 1). Second, when the auditory signal was lateralized, the sound’s spatial information (on the same or opposite side as the visual target) influenced the perceived visual offset position; the visual offset position tended to be attracted toward the side of the sound presentation (Experiment 2). Third, the spatial information of the lateralized sound did not influence the judgment of visual offset timing, implying that the effect of the lateralized sound in Experiment 2 was mainly in the spatial domain (Experiment 3). Fourth, the effect of the lateralized sound was different for visual targets starting from the left or right visual field. For a visual target appearing in the left visual field and moving rightward, RM was not observed, and only a sound presented before physical visual offset shifted the perceived visual offset position backward. However, a lateralized sound from the same direction as the visual target shifted the perceived visual offset position toward the side of the presentation of the sound more strongly than the backward shift observed with lateralized sound from the opposite visual field. For a visual target appearing in the right visual field and moving leftward, RM was observed when the auditory signal was presented from the opposite direction after physical visual offset. When the auditory signal was presented before the physical visual offset, RM was not observed, while the backward displacement of the perceived visual offset position was enhanced by sound from the same direction as the visual target (Experiment 2). We interpret these results to mean that the auditory signal may influence the visual offset position of the moving object through both spatial and temporal processes. Temporal information of the auditory signal influenced the perceived offset timing of the visual object as if it truncated the visual trajectory. However, when the auditory signal occurred in the same hemifield as the visual target, enhanced backward displacement was observed relative to when the auditory signal occurred in the hemifield opposite to the visual target.

The results of Teramoto et al. (2010) suggest that the close association between the auditory and visual signals accomplished by onset synchrony is necessary for the presented sound to have an effect on the perceived position of a visual offset. Their results also suggest that a transient auditory signal presented around the moment of visual motion offset has no influence on perceived visual offset position when another sound is presented at the onset of the motion. The findings of present study seem inconsistent with Teramoto et al.’s (2010). One possible source of discrepancy between their findings and ours would be that Teramoto et al. (2010) presented the auditory signals at both the onset and offset of the visual motion, whereas we presented an auditory signal only at or near the offset of the visual motion. The auditory signal at the onset of the motion might start a duration estimation process that may counteract the auditory influence on the visual offset. To address this question, we performed an experiment for a supplementary examination (*N* = 5), presenting a sound at both the onset and offset of the visual motion. However, the same results as in Experiment 1 were again observed [*F*(7,28) = 7.016, *p* < 0.01]. A tendency for larger backward displacement was observed when the sound was presented before the visual target offset. The pattern showed that perceived visual offset positions were not influenced by sound presented after the visual motion offset. Therefore, it seems that the reason why the offset sound does not exhibit its effect in Teramoto et al.’s (2010) study does not result from the sound presented at the onset.

Another source of discrepancy could be differences between the ways of response acquisition. We asked participants to report the visual offset position directly by clicking a mouse, and we observed backward displacement in all experiments, but RM only in Experiment 2B (around 0.2°), whereas Teramoto et al. (2010) measured visual offset by probe judgments and observed robust RM (around 0.3°–0.6°). Previous research has shown that RM is larger when participants report the offset position by pointing with a mouse (Kerzel et al., 2001). This enhancement might result from the separate processes or representations subserving motor actions and cognitive judgments (Goodale and Milner, 1992). While Goodale and Milner’s model suggests that hand movement is not “deceived” by visual illusion, other researchers have indicated that the mental extrapolation that calculates a visual object’s position by analyzing its speed and trajectory occurs in the motor system to a larger degree than in the visual system (Yamagishi et al., 2001; Kerzel, 2003). Therefore more localization errors occur with motor-oriented measurement methods. A response that depends more upon perception-for-action might lead to larger localization errors both when forward and backward displacement occurs. In the present study, backward displacement was induced by transient sounds, and a response depending more upon perception-for-action might allow for a stronger effect of the auditory signal than response depending more upon perception-for-identification on the perceived offset position of the visual stimulus.

Previous research has shown that a transient visual stimulus presented at the moment of visual motion offset affects the perceived offset position of a visual target. Müsseler et al. (2002) presented a visual flash simultaneously with the offset of a moving visual target and asked participants to judge the target’s position when the flash appeared. They observed no RM; rather, the perceived visual offset position was displaced backward compared to the actual visual offset position, similar to our observations. Although the stimulus parameters and procedure were different, their findings point to the possibility that intramodel interaction (the effect of visual transients on visual localization) might be extended into audiovisual interaction. That is, both visual and auditory transient signals presented before the visual motion offset could induce backward displacement of perceived visual offset position. This will be an interesting venue for future investigations.

In addition, when a brief presented stationary visual stimulus was aligned with the final portion of the moving target’s trajectory, memory for the location of the stationary object was displaced in the direction of motion of the moving target (Hubbard, 2008). It was suggested that RM of the moving target influences the representation of the stationary object’s location, and this influence the stationary object being displaced in the direction of the motion of the moving target. It also implied the possibility that a general mechanism coding both location and motion information. Therefore, information of the stationary object and the moving object influence the perceived position of each other.

The auditory system is generally superior to the visual system in terms of temporal perception, and the visual system is generally superior to the auditory system in terms of spatial perception. Therefore, vision can provide more accurate spatial information, while audition can provide more accurate temporal information. The modality precision hypothesis suggests that the modality with the highest precision with regard to the required task tends to be dominant in multimodal interactions (Shipley, 1964; Welch and Warren, 1980, 1986; Spence and Squire, 2003). In the present study, we found that the perceived visual offset position was shifted backward when the auditory signal was presented before the visual offset. This implies that the perceived timing of the visual motion offset was attracted to the presentation timing of the auditory signal, consequently inducing the backward displacement. This is consistent with auditory superiority for temporal perception (e.g., the temporal ventriloquism effect; Vroomen and de Gelder, 2004). On the other hand, our results also suggest that the effect of lateralized sound was spatial rather than temporal, a finding that cannot be explained by the modality precision hypothesis. There seem to exist significant spatial effects from audition to vision, particularly when blurred visual stimuli which are poorly localized are presented (Alais and Burr, 2004). Teramoto et al. (2012) have demonstrated that spatial aspects of sound can modulate visual motion perception, suggesting that visual and auditory modalities influence each other in motion processing. Thus, taken together, our results indicate that auditory information influences visual perception (at least for the perceived position of a visual offset) via both temporal and spatial processes.

Maus and Nijhawan (2009) proposed a dual-process model to explain differences between how the visual system processes the positions of abruptly vanishing objects and gradual disappearing objects. The first process calculates the position of a moving object in the near future by analyzing its speed and trajectory. When the moving object disappears abruptly, the second process modulates the forward displacement. This modulation mechanism relies on accurate spatial information provided by the transient of the abrupt offset of the moving object. A stronger transient leads to more accurate localization of the moving object because it aids position representation by employing the retinal off-transient to win the competition for perceptual awareness. The present findings could be interpreted to mean that the modulation mechanism relies not only on visual information provided by the retinal off-transient, but also on information provided by a transient auditory signal that is temporally and spatially close to the visual motion offset. If the transient auditory signal is firmly associated with the visual motion offset, the neural system also uses temporal and spatial information provided by the auditory signal to modulate possible overshoots. The present study suggests the possibility that the visual system integrates auditory information presented before and after the offset of visual motion.

However, the results of Teramoto et al.’s study was not consistent with Maus and Nijhawan’s account and the present study. In Teramoto et al.’s study, they suggest that the sustained sound during visual motion is necessary for the audiovisual integration to enhance or reduce RM. Conversely, the results of the present observed the effect of a transient auditory signal on perceived visual offset. However, due to discrepancies in experiment paradigm, parameters, and stimuli, it prevents directly comparisons between the present study or Maus and Nijhawan’s account and Teramoto’s et al.’s study. Perhaps the sustained sound influences the audiovisual integration in a different way with the transient sound. This will also be interesting for future investigations.

Nevertheless, signals from different sensory modalities are not combined indiscriminately. We observed the backward displacement mainly when an auditory signal was presented 120 or 80 ms before the actual visual offset. However, we observed that the spatial information of an auditory signal modulated RM only when sound was presented 80 ms after physical visual offset (Experiment 2B). This might imply that the temporal window during which the visual system integrates auditory information is approximately 100 ms before and after visual motion offset. This is consistent with the temporal window of sound-induced illusory flash (Shams et al., 2002) and multisensory integration in superior colliculus neurons in the mammalian brain (Meredith et al., 1987).

In conclusion, a transient auditory signal presented before or after the offset of physical motion of a visual stimulus can modulate the perceived visual offset position. The magnitude of the backward or forward shift depends on the spatial relation between the auditory and the visual stimulus. In order to elucidate the underlying mechanism of these results, future experiments should be conducted to investigate how closely visual and auditory information must correspond and whether the auditory effect on visual offset occurs when the visual object moves toward the peripheral field. In the present experiments, the visual field, motion direction, and sound position were confounded, and therefore we cannot rule out the possibility that the observed effects were induced by a combination of these factors. Further investigations are warranted to address this issue.

## Conflict of Interest Statement

The authors declare that the research was conducted in the absence of any commercial or financial relationships that could be construed as a potential conflict of interest.
